# Chlorella sorokiniana induces mitochondrial-mediated apoptosis in human non-small cell lung cancer cells and inhibits xenograft tumor growth in vivo

**DOI:** 10.1186/s12906-017-1611-9

**Published:** 2017-02-01

**Authors:** Ping-Yi Lin, Ching-Tsan Tsai, Wan-Ling Chuang, Ya-Hsuan Chao, I-Horng Pan, Yu-Kuo Chen, Chi-Chen Lin, Bing-Yen Wang

**Affiliations:** 1 Transplant Medicine & Surgery Research Centre, Changhua Christian Hospital, Changhua, Taiwan; 2 Department of Medical Research, China Medical University Hospital, Taichung, Taiwan; 3 Department of Public Health, School of Public Health, China Medical University, Taichung, Taiwan; 4 Institute of Biomedical Science, National Chung-Hsing University, Taichung, Taiwan; 5 Biomedical Technology and Device Laboratories, Industrial Technology Research Institute, Hsinchu, Taiwan; 6 Department of Food Science, National Pingtung University of Science and Technology, Pingtung, Taiwan; 7 Department of Biotechnology, Asia University, Taichung, Taiwan; 8 Division of Thoracic Surgery, Department of Surgery, Changhua Christian Hospital, Changhua, Taiwan; 9 School of Medicine, Chung Shan Medical University, Taichung, Taiwan; 10 School of Medicine, Kaohsiung Medical University, Kaohsiung, Taiwan; 11 Institute of Genomics and Bioinformatics, National Chung Hsing University, Taichung, Taiwan

**Keywords:** Chlorella sorokiniana, Human non-small cell lung cancer cells, Mitochondrial apoptotic pathway

## Abstract

**Background:**

Lung cancer is one of the leading causes of cancer related deaths worldwide. Marine microalgae are a source of biologically active compounds and are widely consumed as a nutritional supplement in East Asian countries. It has been reported that Chlorella or Chlorella extracts have various beneficial pharmacological compounds that modulate immune responses; however, no studies have investigated the anti-cancer effects of Chlorella sorokiniana (CS) on non-small cell lung cancer (NSCLC).

**Methods:**

In this study, we evaluated the anti-cancer effects of CS in two human NSCLC cell lines (A549 and CL1-5 human lung adenocarcinoma cells), and its effects on tumor growth in a subcutaneous xenograft tumor model. We also investigated the possible molecular mechanisms governing the pharmacological function of CS.

**Results:**

Our results showed that exposure of the two cell lines to CS resulted in a concentration-dependent reduction in cell viability. In addition, the percentage of apoptotic cells increased in a dose-dependent manner, suggesting that CS might induce apoptosis in human NSCLC cells. Western blot analysis revealed that exposure to CS resulted in increased protein expression of the cleaved/activated forms of caspase-3, caspase-9, and PARP, except caspase-8. ZDEVD (caspase-3 inhibitor) and Z-LEHD (caspase-9 inhibitor) were sufficient at preventing apoptosis in both A549 and CL1-5 cells, proving that CS induced cell death via the mitochondria-mediated apoptotic pathway. Exposure of A549 and CL1-5 cells to CS for 24 h resulted in decreased expression of Bcl-2 protein and increased expression of Bax protein as well as decreased expression of two IAP family proteins, survivin and XIAP.

**Conclusions:**

We demonstrated that CS induces mitochondrial-mediated apoptosis in NSCLC cells via downregulation of Bcl-2, XIAP and survivin. In addition, we also found that the tumors growth of subcutaneous xenograft in vivo was markedly inhibited after oral intake of CS.

## Background

Lung cancer is one of the leading causes of cancer related deaths worldwide. In 2012, an estimated 1.8 million new cases of lung cancer were diagnosed, accounting for approximately 13% of all cancer diagnoses [1]. Non–small cell lung cancer (NSCLC) is the most common type of lung cancer and the 5-year survival rate for patients with this disease is about 22% [2]. Current treatments for NSCLC such as surgery, chemotherapy and radiotherapy are not sufficient and the outcomes of treatment remain poor because of drug resistance and toxicity.

Marine microalgae contain several groups of biologically active compounds including proteins, minerals, vitamins, fatty acids, and antioxidants [3]. Chlorella is a unicellular green microalgae and is widely consumed as a nutritional supplement in East Asian countries. Chlorella or Chlorella extracts have been demonstrated to modulate immune response [4, 5], decrease hepatitis C virus viral load [6], and to have anti-cancer effects in human prostate cancer cell lines [7], human lung cancer cells [8], liver cancer cells [9], and human colon cancer cells [10]. However, the underlying molecular mechanisms governing the antitumor activity of Chlorella remain unclear.

Chlorella sorokiniana (CS), a species of green algae of the genus chlorella, has been demonstrated to be effective at inducing immune responses [4]; however, no studies have investigated the anti-cancer effects of CS in NSCLC cells. In this study, we evaluated the anti-cancer effects of CS in two NSCLC cell lines, namely A549 and CL1-5 human lung adenocarcinoma cells, and its effects on tumor growth in a subcutaneous xenograft tumor model. We also investigated the possible molecular mechanisms governing the pharmacologic function of CS.

## Methods

### Cell culture and reagents

The human lung adenocarcinoma cell lines A549 and CL1-5 were cultured in Dulbecco’s modified eagle medium (DMEM, Invitrogen, Rockville, MD) supplemented with 10% fetal bovine serum (FBS, Gibco, Grand Island, NY) and 100 U/ml of penicillin (Gibco, Grand Island, NY) and 100 mg/ml of streptomycin (Gibco, Grand Island, NY). Cultures were maintained at 37 °C in a humidified incubator in an atmosphere of 5% CO_2_.

### Chlorella sorokiniana extract (CSE)

Chlorella sorokiniana, single-cell thermophilic green algae, was provided by International Cryptomonadales Biotechnology (Taiwan). The Chlorella sorokiniana powders were refluxed with distilled water for 1 h. The extract was filtered by N0.5 filter paper (Tokyo, japan) and vacuum concentrated at 60 °C until the solid content of liquid extract was 5%.

### Cell viability assay

The cytotoxic effects of CS cellular viability were assessed using the 3-(4,5-dimethylthiazol-2-y1) -2,5-diphenyltetra-thallium bromide (MTT) assay (Sigma-Aldrich, St. Louis, MO). Cells were plated in 24-well plates for 24 h, after which various concentrations of CS (0–1000 ng/ml) were added and incubated for 24 h. After removing the medium and adding 200 μL of 1 × MTT solution to each well and allowed to incubate for 4 h. The resulting formazan crystals were then dissolved by adding 600 μL DMSO. The spectrophotometric absorbance was measured at 570 nm in a microplate reader (TECAN, Durham, NC).

### Cell lysis and western blot analysis

Whole cell protein was lysed in 2% SDS comprising 10 mM EDTA, 50 mM Tris base, 10% SDS pH 8.0 and boiled at 95 °C for 10 min. Protein concentrations were assessed using a BCA protein assay kit (Pierce, Rockford, IL). Equal amounts of protein were loaded on 10–15% SDS PAGE gels, transferred to polyvinylidene difluoride (PVDF, GE Healthcare, Freiburg, Germany) membranes and blocked with 5% nonfat milk in TBST buffer (20 mM Tris-HCl,120mMNaCl, 0.1% Tween 20) for 1 h. Membranes were incubated with various primary antibodies against cleaved caspase-3, cleaved caspase-9, cleaved-8, cleaved-PARP, Bcl-2, Bax, XIAP, survivn, cytochorome c, COX-IV, and β-actin (all from cell signaling, Danvers, MA) at 4 °C overnight. After washing, the blots were incubated with HRP-labeled secondary antibodies (cell signaling, Danvers, MA) for 2 h. The signals on the blots were then developed using the enhanced chemiluminescence system (ECL, Perkin Elmer, Waltham, MA) and analyzed with the Hansor Luminescence Image system (Taichung, Taiwan).

### Mitochondria/Cytosol fractionation

The mitochondrial fraction from the cytosolic fraction of A549 and CL1-5 cells was isolated using a mitochondria/cytosol fractionation kit (Biovision, Mountain View, CA). Cells were harvested by trypsinization and washed twice with cold PBS. Then, all cells resuspended in 500 μL of cytosol extraction buffer and maintained on ice for 10 min. Cells were homogenized on ice for 30 to 50 passes using a Dounce tissue grinder (Biovision, Mountain View, CA) and centrifuged at 3000 rpm for 10 min at 4 °C. The supernatants were collected and centrifuged again at 13,000 rpm for 30 min at 4 °C.

### Cell cycle analysis and sub-G1 measurement

Cells (3 × 10^5^ cells/dish) were plated on 6-cm dishes for 24 h. The cells were then treated with various concentrations of CS for different of time intervals and then collected by centrifugation. The pellets were fixed with 75% ethanol at −20 °C overnight. Cells were then centrifuged and resuspended in 500 μL of PI staining solution comprising 2 mg/mL RNase, 1 mg/mL PI and 5% Triton X-100 maintained at 37 °C for 30 min in the dark. The cells were then analyzed using Accuri 5 flow cytometry (BD Biosciences, San Jose, CA). The percentages of cells distribution of the cell cycle in different phases (sub-G1, G0/G1, S, and G2/M) were calculated using C6 Accuri system software (BD Biosciences, San Jose, CA).

### Annexin V assay

Apoptosis assay was measured using a BioVision annexin V-FITC apoptosis detection kit (BioVision, Mountain View, CA). A549 and CL1-5 cells were implanted onto 6-cm dishes for 24 h and then treated with different doses of CS for 12 h. Cells were harvested by trypsinization and then resuspended in 100 μL of binding buffer. Cell suspensions were incubated with 1 μL of annexinV-FITC and 1 μL of propidium iodide (PI) for 10 min at room temperature in the dark. The cells were assessed immediately by Accuri 5 flow cytometry (BD Biosciences, San Jose, CA). The percentage of apoptotic cells (annexin v+) was calculated with C6 Accuri system software (BD Biosciences, San Jose, CA).

### Mitochondrial membrane potential assay

The mitochondria-specific cationic dye JC-1 (Invitrogen, Carlsbad, CA), which undergoes potential-dependent accumulation in mitochondria, was used to measure membrane potential. When the membrane potential (ΔΨ) is below 120 mV, JC-1 is monomeric and emits green light (540 nm) following excitation with blue light (490 nm). At membrane potentials higher than 120 mV, JC-1 monomer aggregates and emits red light (590 nm) following excitation with green light (540 nm). For the assay, cells were seeded onto 96-well plates and treated with various concentrations of CS for 24 h, followed by staining with 10 μg/ml JC-1 for 30 min at 37 °C. The samples were analyzed immediately using Accuri 5 flow cytometry (BD Biosciences, San Jose, CA). The data were analyzed using C6 Accuri system software (BD Biosciences, San Jose, CA) and displayed in a dot plot of JC-1 red fluorescence (Y-axis) against JC-1 green fluorescence (X-axis).

### In vivo antitumor activity

In total, 1 × 10^7^ CL1-5 cells were subcutaneously injected into the right flank of 16-week-old female BALB/c nu/nu mice. Tumor-bearing mice were subdivided into groups of five mice. Therapy was initiated 10 days after tumor inoculation when the mean tumor volume was 50 mm^3^. CS was dissolved in ddH_2_O and mice were fed a daily dose of 50 mg CS/kg body weight. Control mice were fed orally with 100 μL of water. Tumor volumes were calculated as (width)^2^ x length/2 and expressed in mm^3^.

### Statistical analysis

Statistical analyses were performed by using GraphPad Prism software, version 4.0 (GraphPad Software, Inc., La Jolla, CA, USA). Results are expressed as mean ± standard deviation. Statistical analyses were performed by one-way ANOVA followed by Tukey’s post-hoc test. The differences between two groups were compared using the Student’s *t* test. A P-value <0.05 was considered to represent statistical significance.

## Results

### Cytotoxic and cell viability effects of CS in A549 and CL1-5 cells

**Fig. 1:**
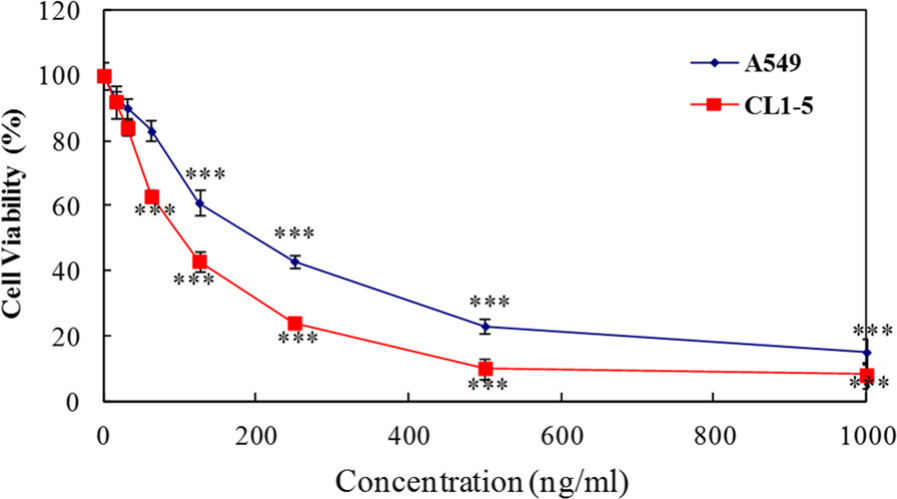
Effects of Chlorella sorokiniana (CS) on viability of A549 and CL1-5 cells. Cells were treated with the indicated concentrations of CS for 24 h following attachment. Cell viability was assessed by the MTT assay. The viability of untreated cells (control) was considered 100%. Each point on the graph represents the mean ± SD of triplicate wells. The data presented are representatives of three independent experiments with similar results. ***P value <0.001 compared with the control group

To determine the cytotoxic effects of CS on cells, A549 and CL1-5 cells were treated with 15.625 to 1000 ng/ml CS for 24 h and then cell viability was determined using the MTT assay. As shown in Fig. 1, exposure of the two cell lines to CS resulted in a concentration-dependent reduction in cell viability.

### CS induces apoptosis in A549 and CL1-5 cells

**Fig. 2:**
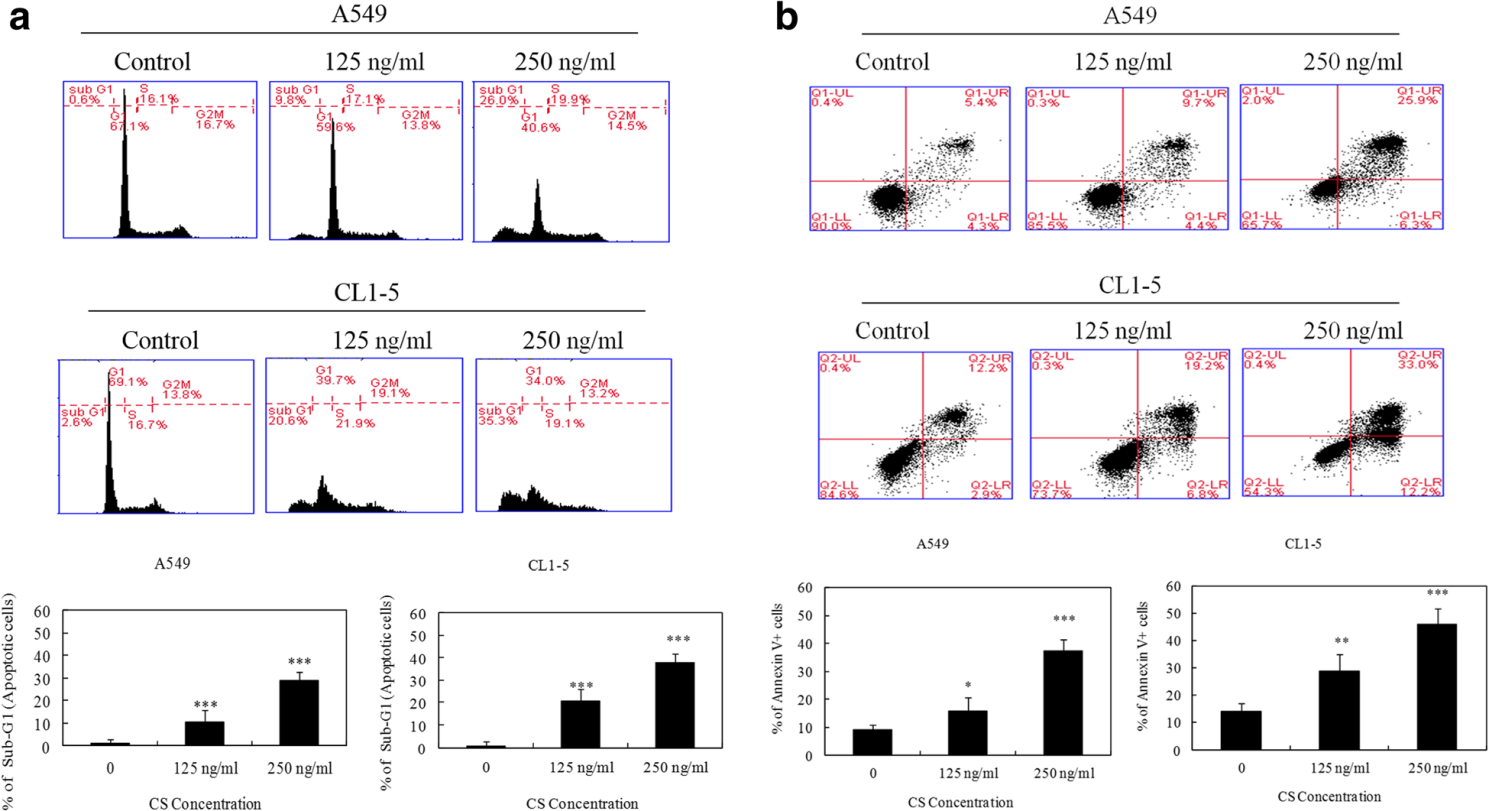
Effects of CS on cell-cycle distribution and apoptosis in A549 and CL1-5 cells. **a** Cell-cycle analysis of CS-treated cells. Cells were treated with the indicated concentrations of CS for 24 h and then subjected to cell cycle analysis. **b** Flow cytometry analysis of CS-induced apoptosis in A549 and CL1-5 cells. The cells were treated with the indicated concentrations of CS for 24 h and then subjected to Annexin V/PI staining. The means ± SD of the experimental triplicates are presented in the bar graph. All data are representative of three independent experiments with similar results. *P value <0.05, **P value <0.01, ***P value <0.001 compared with the control group

To examine whether CS causes cell growth inhibition by inducing cell-cycle arrest or apoptosis, A549 and CL1-5 cells were assayed using PI staining and subjected to flow cytometric analysis. The results are presented in Fig. 2a. No cell cycle arrest was noted after 24 h of exposure to CS; however, there was a significant dose-dependent increase in the number of cells in the sub-G1 phase, which is typically considered to indicate apoptosis. To further determine whether CS induced apoptosis, we used flow cytometry after staining with annexin V-FITC and propidium iodide (PI). As shown in Fig. 2b, the percentage of apoptotic cells (annexin-V+/PI- and annexin V+/PI+) increased in a dose-dependent manner, suggesting that CS might induce apoptotic cell death in human NSCLC cells.

### CS induces caspase-dependent cell death in A549 and CL1-5 cells

**Fig. 3:**
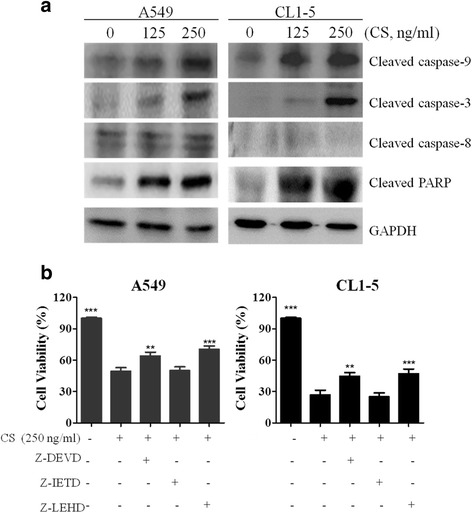
Effects of CS on caspase activation in A549 and CL1-5 cells. **a** Cells were treated with the indicated concentrations of CS for 24 h. Total cell proteins were extracted and immunoblotted with antibodies to detect the cleaved forms of caspase-8, caspase-9, caspase-3, and PARP. **b** Cells were treated with CS (250 ng/ml) and/or the indicated caspase inhibitor for 24 h, and then cell viability was analyzed by the MTT assay. The means ± SD of the experimental triplicates are presented in the bar graph. All data are representative of three independent experiments with similar results. **P value <0.01, ***P value <0.001 compared with the control group

Chemotherapeutic agents can elicit cell death via one of two apoptotic signal transduction pathways, namely an intrinsic (mitochondria-mediated) or extrinsic pathway. These pathways converge at several downstream points, including caspase-3, and/or caspase-7. Activated caspase-3 and/or caspase-7 cleave poly (ADP-ribose) polymerase (PARP), which eventually leads to apoptosis [11]. Thus, in order to clarify the type of a CS-induced apoptotic pathway, the cleaved forms of caspase-8, caspase-9, caspase-3 and PARP were measured by Western blotting. As presented in Fig. 3a, the protein expression of the cleaved/activated forms of caspase-9, caspase-3, and PARP, but not caspase-8, were increased in both cell lines after exposure to CS for 24 h. Activation of caspase-9 and caspase-3 proteins suggests that the mitochondrial pathway is involved in apoptosis. Besides, we used various caspase inhibitors to further confirm our finding. As showed in Fig. 3b, the specific caspase 8 inhibitor, Z-IETD was insufficient to increase cell viability, thereby excluding the possibility of involvement of the extrinsic pathway in CS-induced apoptosis. However, ZDEVD (caspase-3 inhibitor) and Z-LEHD (caspase-9 inhibitor) were sufficient at maintaining cell viability, implying that the mitochondria-mediated apoptotic pathway was the mechanism through which CS induced cell death.

### CS caused loss of mitochondrial membrane potential and cytochrome C release

**Fig. 4:**
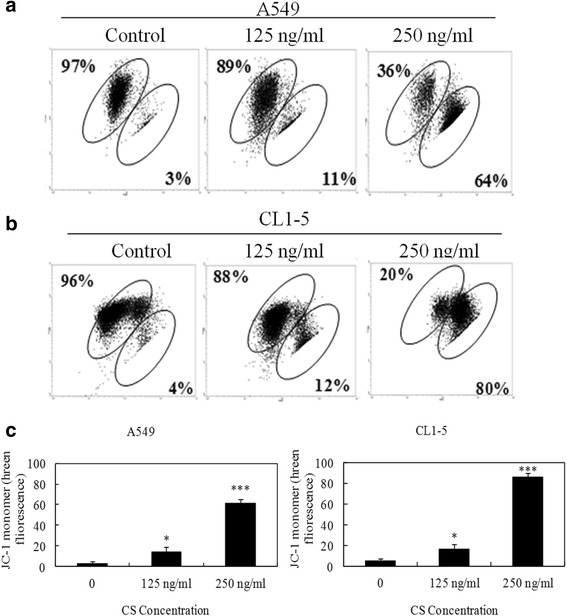
Effects of CS on mitochondrial membrane potential in A549 and CL1-5 cells. **a** and **b** Cells were treated with the indicated concentrations of CS for 24 h and then subjected to JC-1 fluorescence dye staining. The change in mitochondrial membrane potential (ΔΨ*m*) was examined by flow cytometry. **c** The means ± SD of the experimental triplicates are presented in the bar graph. Data are representative of three independent experiments with similar results. *P value <0.05, ***P value <0.001 compared with the control group

**Fig. 5:**
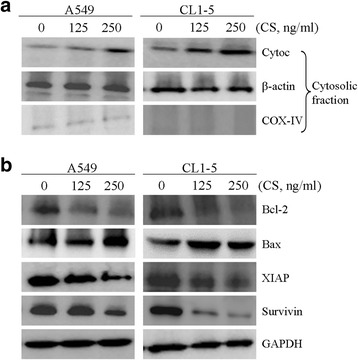
Effects of CS on the expression of cytosolic cytochrome C, Bcl-2 family and IAP proteins in A549 and CL1-5 cells. Cells were treated with the indicated concentrations of CS for 24 h. **a** Cytosolic fractions were extracted and subjected to immunoblotting with anti-cytochrome c antibody. **b** Total cell proteins were extracted and immunoblotted with antibodies to detect Bcl-2, Bax, XIAP, and survivin. All data are representative of three independent experiments with similar results

To confirm whether the mitochondria-mediated apoptotic pathway was involved in the process of apoptosis, the mitochondrial membrane potential (Δψm) in CS-treated A549 and CL1-5 cells was measured. Loss of mitochondrial membrane potential is an indicator of mitochondrial damage during the process of apoptosis. We used the fluorescent cationic dye JC-1 to evaluate the effect of CS on membrane potential. A dose-dependent decrease was observed in red fluorescence in both cell lines, suggesting that CS treatment led to a reduction in Δψm (Fig. 4a). A loss of Δψm can lead to cytochrome c release from the mitochondria into the cytosol, an important event for apoptosis induction [12]. Thus, the cytosolic fractions of A549 and CL1-5 cells treated with 125 and 250 ng/ml of CS were prepared and analyzed for cytochrome c release by Western blotting. As presented in Fig. 5a, the expression of cytochrome c in the cytosolic fractions was observed in both A549 and CL1-5 cells treated with CS. Taken together, our findings show that CS induces cell death via the mitochondrial-mediated pathway.

### CS reduced Bcl-2, Bcl-xl and IAPs protein expression in A549 and CL1-5 cells

Bcl-2 family members are regulators of cytochrome c release from mitochondria during the process of apoptosis [13]. We assessed the protein expression of Bax, a proapoptotic member of the Bcl-2 family that triggers cytochrome c release, and Bcl-2, an antiapoptotic member of the family that inhibits cytochrome c release. As shown in Fig. 5b, CS treatment for 24 h resulted in decreased the levels of Bcl-2 protein and increased the expression of Bax protein. In addition to the Bcl-2 family, the IAP family of proteins also regulates caspase activity, thus affecting apoptosis [14]. Therefore, we measured the expression of two IAP family proteins, namely survivin and XIAP. As demonstrated in Fig. 5b, the levels of XIAP and survivin protein were markedly reduced after CS treatment, particularly in the A549 and CL1-5 cell lines.

### CS inhibits CL1-5 tumor growth in a subcutaneous xenograft tumor model

**Fig. 6:**
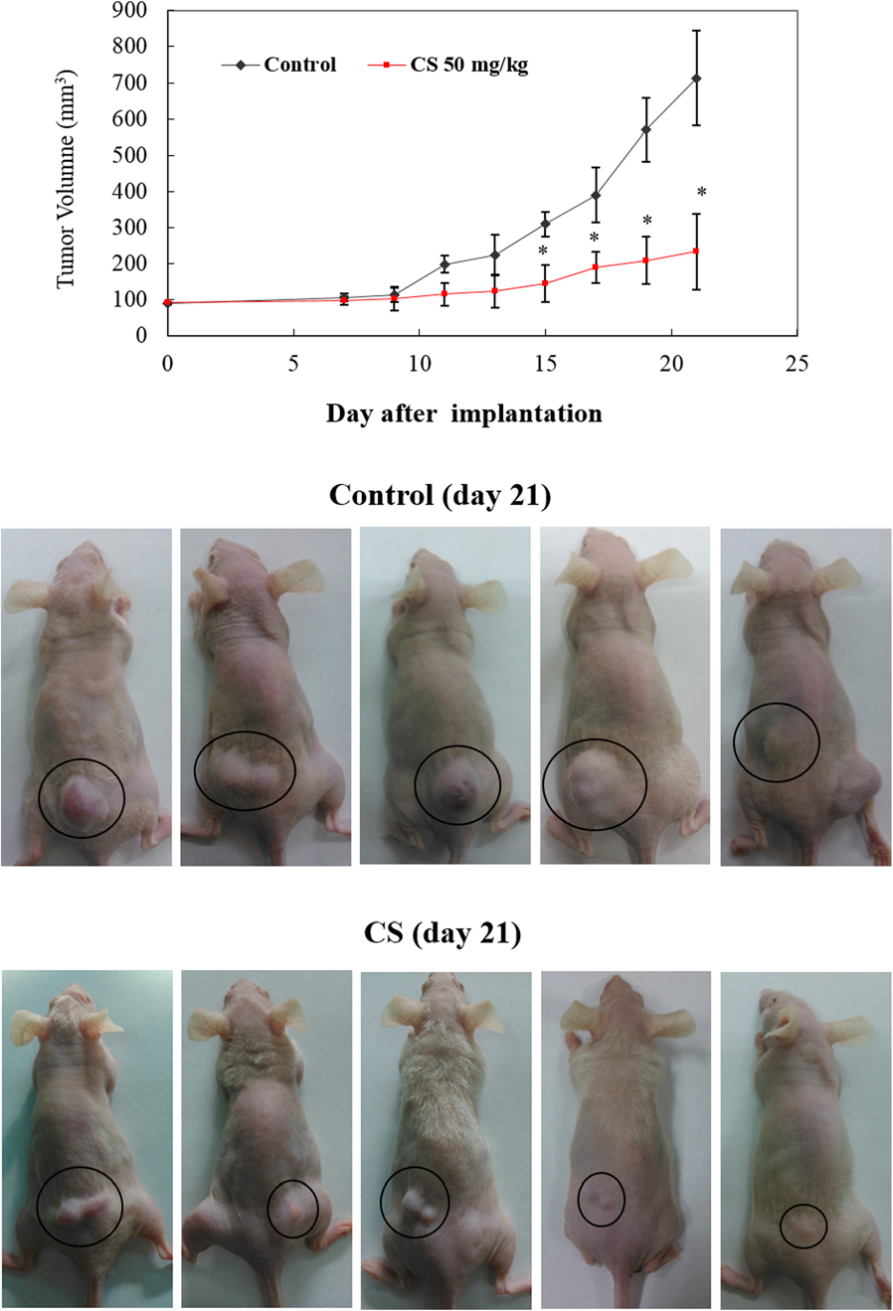
Effects of CS on CL1-5 xenograft tumor growth in vivo. **a** Mean tumor volume was measured at the indicated days after tumor implant. **b** Representative images of mice containing CL1-5 xenografts on day 21. Experiments were repeated two times and provided similar results

The prominent inhibitory effect of CS on NSCLC cell proliferation in vitro suggested that CS might suppress tumor growth in vivo. To verify this hypothesis, female BALB/c nu/nu mice were subcutaneously inoculated with CL1-5 cells and then fed orally with CS starting on day 10 after tumor inoculation. As shown in Fig. 6a, CS at 50 mg/kg resulted in a marked reduction in tumor volume. Representative images of mice containing CL1-5 xenografts on day 21 are presented in Fig. 6b. These results indicate that CS also inhibits the growth of NSCLC cells in vivo.

## Discussion

Several species of Chlorella have been reported to have anti-cancer effects in various cancer cell lines. The most commonly studied Chlorella species are Chlorella vulgaris and Chlorella ellipsoidea. C. vulgaris has been shown to have anti-cancer effects by inducing apoptosis signaling cascades in the hepatoma cell line HepG2 [15] and in rat models of liver cancer [16]. A study comparing the anti-proliferative effects of C. ellipsoidea with those of C. vulgaris in human colon cancer cells revealed that the apoptosis-inducing effect of C. ellipsoidea extract was almost 2.5 times stronger than that of C. vulgaris extract [10]. However, to the best of our knowledge, no studies have explored the anti-cancer effects of C. sorokiniana (CS). The chemical constituents of CS contain three liposoluble fractions and five compounds namely, (22E, 24R)-5alpha, 3beta-epidioxiergosta-6, 22-dien-3beta-ol, (24S)-ergosta-7-en-3beta-ol, loliolide, stigmasta-7, 22-dien-3beta, 5alpha, 6alpha-triol, and 3beta-hydroxy-5alpha,6alpha-epoxy-7-megastigmen-9-one [17]. In our study we found that CS treatment resulted in an increase in the Bax/Bcl-2 ratio and a decrease in expression of IAP family proteins XIAP and survivin in human NSCLC cells. We also found that oral feeding of CS resulted in a marked reduction in tumor volume in a xenograft tumor model in mice. Our results, therefore, demonstrate that CS induces apoptosis in vitro and in vivo.

The anti-cancer effects of some natural compounds have been shown to be the result of their ability to modulate apoptosis signaling pathways [18]. In this study, flow cytometric analysis of PI-labeled cells revealed that exposure to CS resulted in a significant increase in percentage of cells showing elevated hypodiploid DNA content (sub-G1) (Fig. 2a), indicating that CS induces apoptosis in A549 and CL1-5 cells.

Cells undergo cell death through two major signaling pathways, namely the mitochondria-dependent pathway (intrinsic pathway) or the cell surface death receptor signaling pathway (extrinsic pathway) [19]. Caspases play important roles in the regulation of cell death and inflammation [20]. In our study, the presence of early apoptotic cells (annexin V+/PI−) (Fig. 2b), activated forms of caspase-9 and caspase-3, and PARP cleavage indicated that apoptosis (Fig. 3a), rather than necrosis, was involved in CS-induced A549 and CL1-5 cell death.

Bcl-2 family proteins are involved in mitochondrial dysfunction and participate in the mechanism of chemotherapy resistance [21]. The loss of mitochondrial membrane potential is associated with apoptosis via regulation of Bcl-2 family members. The Bcl-2 family comprises anti-apoptotic proteins such as Bcl-2, XIAP, and survivin, and pro-apoptotic proteins such as Bad, Bax, and Bid [13]. Moreover, IAPs (inhibitors of apoptosis) can inhibit apoptosis by directly binding to activated effector caspases, such as caspase-3 and caspase-7, and also inhibit mitochondrial cytochrome c-induced caspase-9 activation [14, 22]. Cytochrome c release from mitochondria into the cytosol acts as a caspase activator and participates in the mitochondrial apoptotic pathway [23]. In this study we identified apoptotic cells by measuring cytochrome c release using a cytometric technique with fluorescence microscopy. The release of this mitochondrial component was labeled by the staining pattern (Fig. 5a). Our results proved that CS induces mitochondrial-mediated apoptosis in human NSCLC cells.

## Conclusions

In conclusion, we demonstrated that caspase-dependent mitochondrial dysfunction is the mechanism through which CS induces apoptosis in NSCLC cells. Downregulation of Bcl-2, XIAP and survivin suggests that CS increases the susceptibility of NSCLC cells to apoptosis induction. In addition, our data show that the growth of subcutaneous xenograft tumors was markedly inhibited by oral administration of CS.

## Abbreviations

CS: Chlorella sorokiniana
CSE: Chlorella sorokiniana extract
NSCLC: Non-small cell lung cancer
PARP: Poly (ADP-ribose) polymerase
